# Government capabilities as drivers of performance: path to prosperity

**DOI:** 10.1016/j.heliyon.2019.e01180

**Published:** 2019-02-04

**Authors:** Nicos Antoniades, Perry Haan

**Affiliations:** a The City University of New York (CUNY)/College of Staten Island (CSI), USA; b Tiffin University, USA

**Keywords:** Business, Economics, Political science

## Abstract

The purpose of this study is to test the relationship between specific capabilities of Cyprus' government and performance and the impact of performance on Cypriots' prosperity. Via the resource-based view model (RBV), it was hypothesized that each capability (i.e., entrepreneurship, motivation, investment, and adaptation) is positively related to Cyprus' government achievement of higher performance. It was also hypothesized that performance is positively related to prosperity. Data collected from 200 Cypriot citizens, aged 18 or over. Using correlation analysis, the study shows that entrepreneurial and adaptive capabilities have a statistical strong positive relationship with performance. In turn, performance has a strong positive relationship with prosperity. Several implications can be drawn from this study's findings for democratic governments and interesting directions for future research are provided.

## 1 Introduction

This study aims to upgrade the political marketing theory by measuring the relationship between specific government capabilities and performance, and the relationship between performance and prosperity via the resource-based view model (RBV). The researchers examined the correlation between specific capabilities (i.e., Entrepreneurship, Motivation, Investment, and Adaptation) and Performance. They also examined the correlation between Cyprus' Government Performance and its people's Prosperity.

The current research not only advances our understanding and debate within the topic area, but it also provides democratic governments with an example and structure of how to deconstruct specific dynamic capabilities in order to achieve higher political performance and lead their country to prosperity. The researchers fill this research gap and open-up great opportunities for new studies that could dramatically upgrade this important area of political marketing. This paper moves on pre-existing literature and builds a conceptual framework. The methodology behind this quantitative survey follows. The paper proceeds with the analysis and presentation of the results and closes with discussion, conclusions, further implications, and suggestions for future research.

## 2 Background

### 2.1 Political marketing and resource-based view

Political marketing is a way politicians use marketing tools and concepts to understand, respond to, involve and communicate with their political markets to achieve their goals. It includes candidates, politicians, leaders, parties, governments, government departments and programs, NGOs, and interest groups (Lees-Marshment, 2014). Marketing concepts are quite applicable to political marketing (Shama, 1975). One such concept is the resource-based view (RBV). According to Penrose (1959), the RBV is a basis for the competitive advantage of a firm and lies primarily in the application of a bundle of valuable tangible or intangible resources (and capabilities) at the firm's disposal.

In 2008, 2009, and 2010, very important attempts were made by scholars to apply the RBV in the political parties' performance (Lynch et al., 2008; O'Cass, 2009; Voola and O'Cass, 2010). Additionally, a recent study expanded the RBV theory by examining specific resources and capabilities as drivers of the U.S. elected politicians' performance (Antoniades and Haan, 2018). However, still more research is necessary to effectively address critical issues relevant to the topic. As shown in Table 1 (Research Gaps), although the above-mentioned studies made a big step to apply the resource-based view model in the political arena, they neglected its importance in the political marketing of a government. What capabilities does a government need to perform effectively? Do these capabilities lead to people's prosperity? Drawing from the literature of marketing and strategic management (Table 1), the current study attempts to fill this important gap in the political marketing literature by discussing the role of specific capabilities in the achievement of a government's performance as a path to its people's prosperity. Antoniades and Haan's model (2018) became an example to build the questions of this study and to transfer the theory to the political marketing of a democratic government. In the following paragraphs, the literature relevant to the subject is reviewed.

**Table 1:** Research gaps.

Research via RBV	RBV & Business	RBV & Political Parties	Articles relevant to Government Performance & Prosperity (Non-RBV)	RBV & Politicians (as units) -	RBV & Government
Entrepreneurial Capability	Holcomb et al. (2009) Audretsch et al., (2002)	Gap	Cavalluzzo and Ittner (2004) Ingraham et al. (2003)	Gap	Gap*
Motivation Capability	Wang et al. (2011) Rueda and Moll (1994)	Gap	Barbuto and Story (2008) Francois (2000)	Gap	Gap*
Investment Capability	Tseng et al. (2012) Asiedu (2006)	Gap	Foggia (2018) Isham et al. (1997) Weaver and Rockman (1993)	Gap	Gap*
Adaptation Capability	Tuominen et al. (2004) Hurley and Hult (1998)	Van Biezen (2005)	Johnson and Arunthanes (1995) Bryer (2011)	Antoniades and Haan (2018)	Gap*

Competitive Adv. & Political Performance	Greene et al., 2015 - Comp. Adv. Leonidou et al., 2012 - Strategy, Competitive Adv., & Performance Banerjee et al., 2003 - Perform. & Competitive Adv.	Voola & O’ Cass (2010) O' Cass (2009) Lynch et al. (2008)	Superior Performance	Superior Performance	Superior∗ Performance

Prosperity∗	Hammond (2017) Fodor (2012)	Gap	Silva et al. (2018)	Gap	Gap*

∗ Gap(s) filled by this study.

### 2.2 Performance

Performance measurement is the process of collecting, analyzing and/or reporting information regarding the performance of an individual, group, organization, system or component (Upadhaya et al., 2014). Performance has to be representative of a particular political stand; it must engage the audience that is its particular target; it should satisfy the formal rules, rituals, and conventions of the institutions through which the meaning is being projected; and be received as logical and coherent (Rai, 2015). Constraints may be transformed into resources that benefit a leader's performance (Helms, 2017). According to Antoniades and Haan (2018), political performance is the degree of citizens'-voters' satisfaction and loyalty, and a politician's reputation.

### 2.3 Prosperity

Prosperity often encompasses wealth but also includes other factors which can be independent of wealth to varying degrees, such as happiness and health. People think about success as the extent to which one's life meets societal expectation, i.e., marriage and children, physical property, and wealth (Silva et al., 2018). In regards to economic growth, it is often seen as essential for economic prosperity, and indeed is one of the factors that is used as a measure of prosperity. Growth rates alone do not tell us much about prosperity; economists generally resort to comparing economic well-being in terms of per capita income, unemployment rate, and poverty rate (Hammond, 2017; Fodor, 2012).

### 2.4 Capabilities

Due to limited research to adequately apply the resource-based view (RBV) in the political arena, the researchers identified a number of organizational capabilities from the business sector with an aim to transfer these capabilities to the political marketing of democratic governments.

#### 2.4.1 Entrepreneurial capability

In economics, an entrepreneur is an entity which has the ability to find and act upon opportunities to translate inventions or technology into new products. The entrepreneur is able to recognize the commercial potential of the invention and organize the capital, talent, and other resources that turn an invention into a commercially viable innovation (Audretsch et al., 2002, 157). Managers are a potential source of value creation for the firm and the managerial ability affects positively resource productivity and increases the quality of firm resources (Holcomb et al., 2009). Government management and elected officials who use resources more carefully and strategically have a better chance to achieve superior performance (Ingraham et al., 2003); government's top management commitment to the use of performance information has a significant positive effect on its performance (Cavalluzzo and Ittner, 2004).

#### 2.4.2 Motivation capability

Motivation can be conceived of as a cycle in which thoughts influence behaviors, behaviors drive performance, performance affects thoughts, and the cycle begins again (Rueda and Moll, 1994). Each stage of the cycle is composed of many dimensions including attitudes, beliefs, intentions, effort, and withdrawal which can all affect the motivation that an individual experiences. The ability to motivate leads to performance and innovation (Wang et al., 2001). Barbuto and Story (2008) argued that motivation and government workers' performance have a very strong positive relationship, whereas Francois (2000) supported that public service motivation (PSM) inclines employees to provide effort out of concern for the impact of that effort on a valued social service (Francois, 2000).

#### 2.4.3 Investment capability

Investment behavior is positively related to reliable information that prompts a switch toward sustainable choices (Foggia, 2018). Thus, the investment capability of the firm affects the efficiency and leads directly to the achievement of competitive performance (Tseng et al., 2012). Further to the above, government investment projects increase citizen voice and public accountability through both participation and better governance and lead to greater efficacy in government action (Isham et al., 1997). Lower inflation, good infrastructure, an educated population, openness to Foreign Direct Investment (FDI), less corruption, political stability, and a reliable legal system have a similar effect. Countries that are small or lack natural resources (like Cyprus) can attract FDI by improving their institutions and policy environment (Asiedu, 2006). Examining the ability of Parliamentary governments to respond effectively to economic development, Weaver and Rockman (1993) argued that innovation and implementation of energy policies (as factors affecting investing) have a positive impact on a government's performance.

#### 2.4.4 Adaptation capability

Adaptability has received increased academic attention as both an input and output factor in business processes (Tuominen et al., 2004). Empirical evidence suggests that adaptive ability is a source of both sustainable competitive advantage and success in new product development (NPD) and commercialization (Hurley and Hult, 1998). According to Johnson and Arunthanes (1995), a firm's performance is significantly affected by major drivers of adaptation such as government regulation, infrastructure differences, and market lag.

In another dimension, Van Biezen (2005) argued that the contemporary literature on political parties has made significant progress with regard to elaboration of models of party adaptation but it has failed to confront the challenge of developing theories of party, politicians, and government's formation that can also be applied to cases other than the Western European. Building on social media, Bryer (2011) argued that the ability of government agencies to adapt to social media builds strong relationships with citizens. Last but not least, via the resource-based view tool, Antoniades and Haan (2018) supported that adaptive capability is positively related to a politician's performance.

## 3 Model

Antoniades and Haan (2018) were the first to examine the relationship between specific U.S. elected politicians' capabilities and political performance; they defined adaptation capability as “the ability to adapt to the needs of people”, “the ability to search for innovative ideas for the benefit of the public interest”, and “the ability to adapt to the political and economic environment”. In regards to political performance, Antoniades and Haan (2018) defined it as “the degree of citizens’-voters’ satisfaction and loyalty, and political reputation”. Building on this idea, this study aims to apply specific capabilities to the political marketing of a democratic government and to examine their relationship with performance. Further to adaptation, more capabilities could consist of a government's framework as a path to its performance and its people's prosperity. Thus, entrepreneurial capability, as “a potential source of value creation” was adapted from Holcomb et al. (2009); motivation capability, as a factor that “leads to performance and innovation” was adapted from Wang et al. (2001), and, investment capability, as a factor that “affects the efficiency and leads directly to the achievement of competitive performance” was adapted from Tseng et al. (2012). Finally, prosperity was adapted from Hammond (2017) and Fodor (2012) and represents per capita income, unemployment rate, and poverty rate (Table 2).

**Table 2:** Operationalization of constructs.

**GOVERNMENT PERFORMANCE (GPE)**	Adapted from Antoniades and Haan (2018)
GPE1: I am satisfied with the Government's efforts for the well-being of its people	
GPE2: I believe in what the Government is trying to do for the welfare of its people	
GPE3: I believe that the Government has succeeded in gaining the trust of its people and strengthening its reputation	
**ORGANIZATIONAL CAPABILITIES**	
***H1. There is a positive relationship between Cyprus' Government Entrepreneurial Capability and Performance***	
**Gov. Entrepreneurial Capability (GEC)**	Adapted from Holcomb et al. 2009
GEC1: The Government has the right people to manage and to promote changes to the common interest	
GEC2: The Government is assertive	
GEC3: The Government successfully deals with any crises	
GEC4: The Government successfully addresses issues such as violence, criminality, immorality, inequality, ineffectiveness, etc.	
GEC5: The Government struggles for institutional changes (i.e., equal opportunities, etc.)	
***H2: There is a positive relationship between Cyprus' Government Motivation Capability and Performance***	
**Gov. Motivation Capability (GMC)**	Adapted from Wang et al. (2001)
GMC1: The Government motivates its people to become more active	
GMC2: The Government encourages its people to react and contribute on combating to negative phenomena	
GMC3: The Government encourages its people to become aware of global issues (i.e., wars, climate change, etc.)	
GMC4: The Government motivates young people to engage in politics and encouraging them to participate actively in the drafting of the law to improve society	
***H3.There is a positive relationship between Cyprus' Government Investment Capability and Performance***	
**Gov. Investment Capability (GIC)**	Adapted from Tseng et al. (2012)
GIC1: The Government produces proposals that promote the common interests	
GIC2: The Government has the ability to invest its funds for the common interest	
GIC3: The Government has the ability to find financial resources for the creation of infrastructure and investment projects	
GIC4: The Government has the ability to create infrastructures that enhance Education and Culture	
***H4. There is a positive relationship between Cyprus' Government Adaptation Capability and Performance***	
**Gov. Adaptation Capability (GAC)**	Adapted from Antoniades and Haan (2018)
GAC1: The Government adapts to the needs of its people	
GAC2: The Government looks for innovative ideas for the benefit of the public interest	
GAC3: The Government adapts to the economic and political environment	
**PROSPERITY**	
***H5: There is a positive relationship between Cyprus' Government Performance and its people's Prosperity***	
**PROSPERITY (PRS)**	Adapted from Hammond (2017); Fodor (2012)
PRS1: I am satisfied with the Government's efforts to reduce unemployment	
PRS2: I am satisfied with the Government's efforts to reduce the poverty index	
PRS3: I am satisfied with the Government's efforts to improve productivity and increase per capita income	

Respondents were asked to indicate their perception about the abovementioned capabilities characteristics exercised by Cyprus' government. Each one of these characteristics was measured by seven-point Likert (1932) rating scales ranging from 1 (Strongly Disagree) to 7 (Strongly Agree). Entrepreneurial capability was measured by a five-item, seven-point rating scale; motivation and investment capabilities were measured by a four-item, seven-point rating scale, and finally, adaptation capability, performance, and prosperity were measured by a three-item, seven-point rating scale each. All constructs are presented in Table 2.

## 4 Hypothesis

**Fig. 1:**
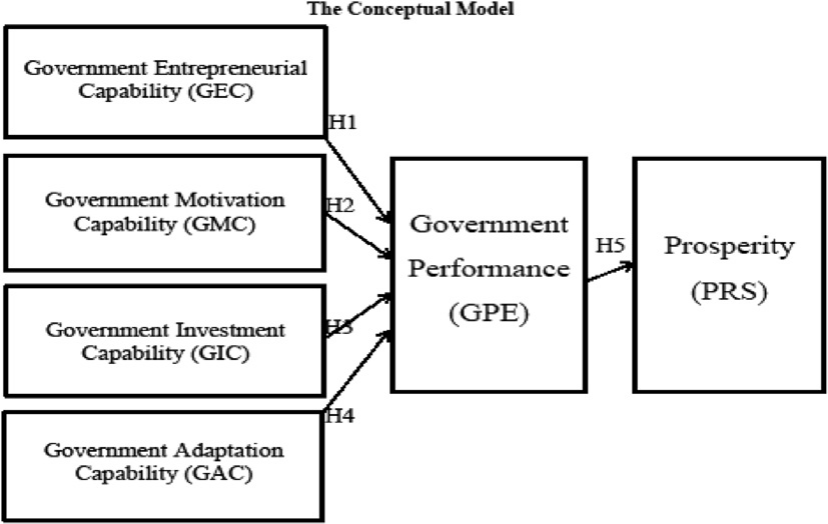
The conceptual model.

Based on the above literature, the conceptual model comprises five hypothesized associations between key constructs (i.e., Entrepreneurial, Motivation, Investment, and Adaptive Capabilities) and performance, and between performance and prosperity (Fig. 1). The researchers created the following hypotheses:

1) ***There is a positive relationship between Cyprus' Government Entrepreneurial Capability and Performance***
2) ***There is a positive relationship between Cyprus' Government Motivation Capability and Performance***
3) ***There is a positive relationship between Cyprus' Government Investment Capability and Performance***
4) ***There is a positive relationship between Cyprus' Government Adaptation Capability and Performance***
5) ***There is a positive relationship between Cyprus' Government Performance and its people's Prosperity***

## 5 Methodology

This quantitative research took place in the Republic of Cyprus, one of the 28 members of the European Union. Cyprus was chosen as the subject of this study due to its 2012–2013 financial crisis and bailout (Dixon, 2013). Cypriot voters who participated in the study completed an online questionnaire voluntarily and anonymously. Therefore, no ethical approval was required by the researchers' institutions (The City University of New York and Tiffin University, Ohio).

A questionnaire link was designed with Survey Monkey online software (Survey Monkey, 2018); Survey Monkey automatically exports data into an Excel spreadsheet. According to the official page of the Cyprus government (Cyprus Gov. 2018), there were 550,876 registered voters in 2018. A total of 1,290 invitations (with a Survey Monkey link) were sent by email. Email addresses were randomly selected from the Cyprus Telecommunications Authority finder (CYTA, 2018). The process resulted in 200 completed questionnaires. The questionnaire was distributed and returned between 10 and 29 March 2018.

The questionnaire used a structured approach with closed statements (based on a 7-point Likert rating scale). Thus, the questions attempted to capture the intensity of the voters' feelings for a given item (i.e., Entrepreneurial Capability). An Excel spreadsheet was copied to RStudio (2015) statistical program for further statistical analysis. The statistics used in this study were as follows: (a) the mean; (b) the standard deviation (Walker, 1931); (c) Pearson (1895) correlations to measure the linear relationship between the variables (Rumsey, 2018), and lastly, statistical hypothesis testing (Wasserstein and Lazar, 2016). The p-value is a number between 0 and 1 and interpreted in the following way: A small p-value (typically ≤0.05) indicates strong evidence against the null hypothesis (Table 3). Statistical results are presented in Table 4.

**Table 3:** Regression analysis.

**Lavaan 0.6–3 ended normally after 21 iterations**				
Optimization method: NLMINB				
Number of free parameters: 7				
Number of observations: 200				
Estimator: ML				
Model Fit Test Statistic: 33.571				
Degrees of freedom: 4				
P-value (Chi-square): 0.000				
**Parameter estimates:**				
Information: Expected				
Information saturated (H1) model: Structured				
Standard Errors: Standard				
**Regressions:**				
	Estimate	Std. Error	z-value	P (>|z|)
GPE ∼				
GEC	0.827	0.085	9.680	0.001
GMC	-0.027	0.078	-0.346	0.729
GIC	-0.046	0.095	-0.486	0.627
GAC	0.418	0.077	5.429	0.001
PRS ∼				
GPE	0.456	0.038	12.142	0.001
**Variances**				
	Estimate	Std. Error	z-value	P (>|z|)
GPE	0.501	0.050	10.000	0.001
PRS	0.884	0.088	10.000	0.001

**Table 4:** Statistical analysis.

	Mean	Cronbach's Alpha	St. Deviation	Pearson Cor. (GPE)	p-value
Government Entrepreneurial Capability (GEC)	3.578	0.95	1.38	0.90	0.001
Government Motivation Capability (GMC)	3.125	0.92	1.27	0.70	0.729
Government Investment Capability (GIC)	3.678	0.94	1.5	0.81	0.627
Government Adaptation Capability (GAC)	3.483	0.92	1.5	0.87	0.001

Government Performance (GPE)	3.573	0.96	1.77		
				GPE (PRS)	0.001
Prosperity (PRS)	3.48	0.96	1.24	0.65	

## 6 Results

### 6.1 Respondents' political orientation

Respondents were asked to answer for their political orientation on a 1–7 scale (where 1, 2 and 3 equal to “left-oriented thinkers”, 4 equals to “center-oriented thinkers”, and 5, 6 and 7 equal to “right-oriented thinkers”). This measurement helped the researchers to have a view of the respondents' political orientation; the average was 4.2 which indicates that the sample was very well balanced as regards the political orientation of the respondents (Table 5).

**Table 5:** Demographic profile.

Group	Participants (%)
**Gender**	
Men	61
Women	39
**Ages**	
18–29	11
30–45	39
46–55	31
56–65	17
65+	2
**Education**	
Less than High School	0
High School Diploma	5
College	25
Bachelor Degree	27
Master/Doctoral Degree	43
**Political Orientation**	
Left-oriented thinkers (1–3)∗	32
Center-oriented thinkers (4)∗	27
Right-oriented thinkers (5–7)∗	41

∗ Based on a 1–7 Likert Scale.

### 6.2 Respondents' demographics

Sixty-one percent of the respondents were men; 39% were women. Ninety-five percent of the respondents were college or university graduates and only 5% were High-School graduates; the very high level of Cypriots' education facilitated their perception of this study. All demographic information is presented in Table 5.

### 6.3 Hypothesis testing

H1 ***There is a positive relationship between Cyprus' Government Entrepreneurial Capability and Performance***.

The correlation value (0.90) shows a very strong positive relationship between Cyprus' Government Entrepreneurial Capability and Performance. The results are significant with a p-value of 0.001.

H2 ***There is a positive relationship between Cyprus' Government Motivation Capability and Performance.***

The correlation value (0.70) shows a strong positive relationship between Cyprus' Government Motivation Capability and Performance. The results are not significant with a p-value of 0.729.

H3 ***There is a positive relationship between Cyprus' Government Investment Capability and Performance.***

The correlation value (0.81) shows a strong positive relationship between Cyprus' Government Investment Capability and Performance. The results are not significant with a p-value of 0.627.

H4 ***There is a positive relationship between Cyprus' Government Adaptation Capability and Performance.***

The correlation value (0.87) shows a very strong positive relationship between Cyprus' Government Adaptation Capability and Performance. The results are significant with a p-value of 0.001.

H5 ***There is a positive relationship between Cyprus' Government Performance and its people's Prosperity.***

The correlation value (0.65) shows a strong positive relationship between Cyprus' Government Performance and its People's Prosperity. The results are significant with a p-value of 0.001.

### 6.4 Further correlation analysis

The researchers attempted to measure the relationship between each one of the abovementioned capabilities and Cyprus' government performance, and the relationship between government performance and prosperity, in specific groups of people; the analysis showed no significant variations in accordance with the overall results (Table 6). This is due to the small size of Cyprus which shows very limited demographic alterations and due to the fact that 37% of the island is occupied by Turkish troops since 1974. As a result of the “Cyprus Problem”, Cypriot politicians, the media, and people follow their “own” political agenda which ends in downgrading economic and other important political issues. However, it is important to mention that right-oriented thinkers' perception in regards to their government's “entrepreneurial”, “investment”, and “adaptation” capabilities, received lower correlation values. Not surprisingly, right-oriented thinkers are generally wealthier than center-oriented and left-oriented thinkers. Thus, entrepreneurial, investment and adaptation capabilities show a much weaker positive relationship with prosperity in right-oriented thinkers (0.26, 0.27, and 0.24 respectively). In regards to motivation capability, right-oriented thinkers seem to realize Cyprus' government weakness to motivate its people; a value of 0.62 shows a relatively strong positive relationship with prosperity.

**Table 6:** Correlation by “Political Orientation”.

Political Orientation	Dependent Variable	Gov. Entrepr. Capability (GEC) Cor.	Gov. Motivation Capability (GMC) Cor.	Gov. Investment Capability (GIC) Cor.	Gov. Adaptation Capability (GAC) Cor.
Left-oriented thinkers (1–3)	GPE∗	0.79	0.64	0.7	0.65
	PRS∗∗	0.58	0.56	0.54	0.45
Center-oriented thinkers (4)	GPE	0.93	0.49	0.66	0.84
	PRS	0.78	0.82	0.69	0.65
Right-oriented thinkers (5–7)	GPE	0.86	0.42	0.81	0.89
	PRS	0.26	0.62	0.27	0.24

∗ Government Performance.

∗∗ Prosperity.

## 7 Discussion

Confirming Audretsch et al. (2002, 157), “entrepreneurial capability” has a very strong positive relationship with a government's performance. The results of this study showed that Cyprus' government has the ability to find and act upon opportunities to translate inventions or technology into new products; the government is able to recognize the commercial potential of the invention and organize the capital, talent, and other resources that turn an invention into a commercially viable innovation.” Cyprus' government seems to realize that its people are a potential source of value creation for the country. Furthermore, its “entrepreneurial ability” also affects positively the country's resource productivity and increases the quality of these resources (Holcomb et al., 2009); especially after the 2013 bailout (Dixon, 2013). As a result, Cyprus' government “performance” facilitates its attempts to lead the country to prosperity; i.e., well-being in terms of per capita income, unemployment rate, and poverty rate (Hammond, 2017; Fodor, 2012).

“Adaptive capability” plays an important role in the achievement of higher government performance confirming Antoniades and Haan's study (2018) who applied adaptation capability in the political marketing of U.S. politicians. Going a step further, this study found that “adaptive capability” not only acts as a driver of a politician's and a government's performance but it can also lead a country to a prosperous “path”. According to the findings of this research, there is a very strong positive relationship between adaptation and performance, and a strong positive relationship between performance and prosperity. Cyprus' government managed to adapt to its people's “needs”; its “adaptive capability” leads to a source of both sustainable competitive advantage and success in new product development (NPD) and commercialization (Hurley and Hult, 1998).

On the contrary, the Cyprus government's weakness to motivate its people seems to affect its performance negatively. The same exists for its “investment capability” that also affects negatively the country's efficiency and inhibits Cyprus' government's attempts to achieve higher performance (Tseng et al., 2012). According to the results of this study, there is a lack of government investment projects that would increase citizen voice and public accountability through both participation and better governance in order to lead to greater efficacy in government action (Isham et al., 1997). Last but not least, more investment “actions” by Cyprus' government would lead to less corruption, political stability, and a more reliable legal system (Asiedu, 2006).

## 8 Conclusions

According to the findings of this study, the Cyprus' government entrepreneurial and adaptive capabilities have a statistically significant very strong positive impact on its performance. In turn, Cyprus' government performance has a statistically sifnificant strong effect on its people's prosperity. Despite their strong positive relationship with performance, Cyprus' government motivation and investment capabilities were not found statistically significant.

This study addresses the paucity of research that operationalizes capabilities as drivers of a democratic government's performance that leads to prosperity and provides practitioners and academics within and beyond the context of political marketing a mechanism to understand the importance of measuring a government's performance via the resource-based view tool. This case not only advances our understanding and debate within the topic area, but it also provides political stakeholders with an example and structure of how to deconstruct specific dynamic capabilities in order to achieve political performance and lead a country to prosperity. This understanding will support the development of political marketing in terms of leadership approach; it will also support the long-term strategic marketing management process. This research may also be used by scholars and political specialists as a basis to explore more dynamic capabilities; it may consist of a more comprehensive framework that focuses not only on “entrepreneurial” and “adaptive” capabilities but on a government's assessment of the opportunities and citizens' needs with an aim to lead its country to “prosperity”. This study sheds light on the general theory of political marketing and moves it to a stronger theory. It also facilitates democratic governments to get to know their resources and capabilities with an aim to achieve higher performance. Most importantly, it consists of a valuable framework of what voters-citizens need from their government; it is a barometer between a government's performance and its people's prosperity.

## 9 Related work

The resource-based view tool could open-up great opportunities for political marketing. New studies can adapt this study's model (Entrepreneurial, Motivation, Investment, and Adaptation capabilities as drivers of performance and prosperity) in order to build on the political marketing of political parties and individual politicians (Table 1). Further research could identify new government resources and capabilities and collect data from other democratic governments. This study focused on a small country and this may limit the generalizability of the findings to other countries; future research could validate the findings using data obtained from much bigger countries (e.g., the U.S.A., or the United Kingdom, etc.).

## Declarations

### Author contribution statement

Nicos Antoniades: Conceived and designed the experiments; Performed the experiments; Analyzed and interpreted the data; Contributed reagents, materials, analysis tools or data; Wrote the paper.

Perry Haan: Conceived and designed the experiments; Analyzed and interpreted the data; Contributed reagents, materials, analysis tools or data; Wrote the paper.

### Funding statement

This research did not receive any specific grant from funding agencies in the public, commercial, or not-for-profit sectors.

### Competing interest statement

The authors declare no conflict of interest.

### Additional information

No additional information is available for this paper.
